# Preparing Italian residents for global medical practice: the role of internationalization in education

**DOI:** 10.1186/s41077-025-00394-8

**Published:** 2025-11-25

**Authors:** Claudia Ebm, Cherrelle Smith, Manuela Milani, Mia Karamatsu, Nick Pokrajac, Bernard Dannenberg, Maurizio Cecconi

**Affiliations:** 1 Department of Biomedical Sciences, Humanitas University, Via Rita Levi Montalcini, 4, Pieve Emanuele, Milan, 20072 Italy; 2 Department of Emergency Medicine, Stanford University School of Medicine, Palo Alto, CA USA; 3 IRCCS Humanitas Research Hospital, Rozzano, Milan, Italy

**Keywords:** Simulation-based education, Internationalisation, Intercultural competence, Faculty exchange, Postgraduate medical education

## Abstract

**Background:**

As medical education becomes increasingly global, there is a need to prepare residents for culturally diverse clinical environments. Key questions remain about how best to define, measure, and demonstrate the achieved benefits of international education initiatives, particularly regarding intercultural competence and global adaptability for healthcare professionals. This study addresses this gap by assessing a hybrid educational model that combines international faculty mobility with simulation-based learning.

**Methods:**

We implemented the Paediatric EmergenSIMs Pathway, where visiting U.S. faculty led high-fidelity pediatric emergency simulations and culture-focused lectures, followed by debriefings. A cross-sectional mixed-methods design was used, including post-course surveys, interviews, and a separate faculty survey. Quantitative data were analyzed descriptively; qualitative data underwent thematic analysis.

**Results:**

Thirty-five participants (70% response rate) completed the survey. Residents reported learnings of cultural awareness (M = 4.72, 95% CI [4.66–4.78]) and cultural competency (M = 4.65, 95% CI [4.54–4.75]), with slightly lower but still positive learnings in cultural sensitivity (M = 4.08, 95% CI [3.93–4.23]). Qualitative feedback confirmed that international faculty and simulations broadened cultural perspectives and fostered social skills; faculty noted mutual learning and challenges in sustaining partnerships.

**Conclusions:**

Integrating international faculty and simulation-based education fostered intercultural competence, reflective practice, and professional growth. The hybrid format enabled safe, experiential learning and mutual exchange. These findings highlight the need for standardized frameworks to assess intercultural learning linked to faculty mobility and to inform future global medical curricula.

**Supplementary Information:**

The online version contains supplementary material available at 10.1186/s41077-025-00394-8.

## Key messages

• Internationalization enriches medical training with global perspectives and cross-cultural skills.

• Hybrid educational models, combining simulation and international faculty exchange, enhance learning and global collaboration.

• Standardized and pragmatic evaluation frameworks are needed to measure transferable outcomes.

**Supplementary Information:**

The online version contains supplementary material available at 10.1186/s41077-025-00394-8.

## Introduction

The internationalisation of medical education (IoME) has become a cornerstone in preparing physicians for an increasingly interconnected world. Modern healthcare professionals must not only deliver safe and evidence-based care but also act with cultural sensitivity, ethical awareness, and adaptability across diverse health systems [1]. As patient populations become more heterogeneous and global mobility intensifies, medical curricula must evolve beyond the transmission of technical and cognitive skills to intentionally cultivate intercultural and ethical competence [2, 3].

Central to this capability is cultural competence, which encompasses cultural awareness (recognizing how culture shapes beliefs and care), cultural sensitivity (openness and respect), and the ability to apply this understanding effectively in clinical practice [4, 5]. Historically, medical education has prioritized procedural skills, while intercultural learning has often been informal or peripheral [1, 3, 6]. Yet, the ability to navigate cultural diversity is now essential for patient-centred care. Effective training strategies must therefore make intercultural learning tangible, measurable, and transferable [7].

Two approaches have gained particular attention in this respect: faculty mobility and simulation-based education. Faculty mobility enables educators from different cultural and professional contexts to exchange perspectives, enriching learners’ understanding of varied clinical practices and healthcare philosophies [8, 9]. Simulation-based education offers a psychologically safe, realistic setting to practise intercultural interactions, from communication barriers to culturally influenced decision-making [10–12]. Together, these approaches create a structured environment where learners can actively apply and reflect on new knowledge and skills before entering real-world clinical practice.

However, both international faculty exchanges and simulation-based programs require substantial investment in time, personnel, and resources. For their continued implementation, it is essential to demonstrate not only their educational benefits but also their clinical and systemic value [13, 14]. Despite the growing enthusiasm for IoME, there remains no shared framework to define, measure, or evaluate benefits -or value achieved- particularly regarding intercultural outcomes linked to faculty mobility [15, 16].

To address this gap, we developed and implemented the Paediatric EmergenSIMs Pathway, an international, simulation-based training initiative co-created by Humanitas University (Italy) and the paediatric emergency department of Stanford University (USA). The program introduced visiting international faculty with extensive educational experience in a system where intercultural and global dimensions are integral to training [17], into Italian residency programs. This collaboration provided structured exposure to global educational practices and simulation-based learning, enhancing participants’ cultural sensitivity, teamwork, and readiness for global clinical practice. Beyond its immediate learning outcomes, this initiative aims to contribute to a broader conversation about how benefits of international faculty mobility and simulation-based education can be systematically integrated into postgraduate medical training to foster intercultural competence and prepare residents for globalized healthcare environments.

## Methods

### Study design and context

**Fig. 1:**
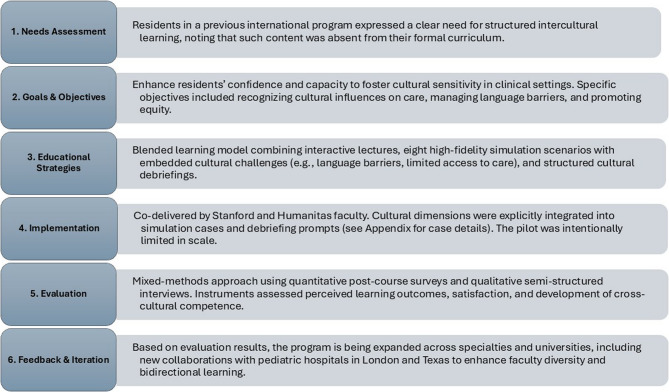
Illustrating the application of Kern’s framework

This study employed a cross-sectional mixed-methods design to evaluate residents perceived intercultural learning following participation in an internationalized, simulation-based educational program. The study was conducted within the *Paediatric EmergenSIMS Pathway*, an educational program at Humanitas University, Italy. The program incorporated a faculty exchange model in which visiting instructors actively participated in simulation-based teaching, debriefing, and reflective discussions alongside local faculty. As shown in Fig. 1, curriculum development followed Kern 6-Step Framework [18], and our internal project management approach was used to assess and track the success of the initiative [19].

### Participants

All third- to fifth-year anaesthesiology and emergency medicine residents at Humanitas University were invited to participate. To enrich group discussions with diverse perspectives, fifth-year medical students, as part of their paediatric curriculum components, and nurses were also invited. Demographic data, including training year, prior international experience, and language proficiency, were collected to contextualize the findings.

### Educational intervention

The Paediatric EmergenSIMs Pathway comprised eight high-fidelity simulation scenarios with debriefing; replicating critical paediatric emergencies. Each case was designed to address both clinical and non-clinical learning objectives as described in Table 1 [5]. Each session included a structured debriefing co-facilitated by all faculty members, with a deliberate focus on communication styles, cultural values in team dynamics, and adaptability to differing professional norms.

**Table 1:** Definitions and examples of the delivery and assessment of intercultural competences

Domain	Definition	Simulation Case Examples	Survey Item Examples
Cultural Awareness	Recognizing how cultural norms shape communication, decision-making, and care	• Paediatric patient with a non-local guardian • End-of-life care involving culturally specific rituals	Awareness how cultural beliefs influence how families perceive healthcare. Understanding how healthcare practices vary across different cultures.
Cultural Sensitivity	Demonstrating openness and respect toward diverse values and practices	• Refugee patient with trauma history • Adolescent with language barrier and undocumented status	Appreciation of other cultural norms Cultural perspectives enriched my understanding and behaviour
Cultural Competence	Applying intercultural understanding in clinical practice	• Emergency care for migrant worker • Cross-cultural team handover scenario	Interest to learn about cultures different from my own. Confidence to provide culturally appropriate care.

### Evaluation tool

To assess the impact of the Paediatric EmergenSIMs Pathway, we designed a post-course survey aligned with the program’s intercultural learning objectives. The 13-item questionnaire, validated by faculty, explored residents’ perceptions across three domains and grouped them accordingly. Participants completed the anonymous online survey immediately after the course, which included Likert-scale items and open-ended questions. Faculty completed a separate 22-item survey on their experiences and perceived barriers to international collaboration. For the qualitative phase, six residents (four Anaesthesiology, two Emergency Medicine) were purposively sampled to reflect variation in training and experience. Interview responses were grouped into four predefined categories covering intercultural learning, professional development, barriers, cultural awareness.

### Data analysis

Quantitative data were analysed using descriptive and inferential statistics (mean, SD, median, 95% CI), with Likert responses coded from 1 (Strongly Disagree) to 5 (or 6) (Strongly Agree). Qualitative data from open-ended responses and interviews were analysed thematically by a researcher using a structured coding framework. To enhance methodological rigor and credibility, the coding process was documented in detail, and preliminary themes were reviewed with an external peer for validation.

### Ethical considerations

The study was approved by the Humanitas University Ethics Committee. Participation was voluntary, responses anonymized, and data used solely for research and program improvement.

## Results

### Participant characteristics

Fifty individuals participated in the *Paediatric EmergenSIMS Pathway* (24 residents, 24 medical students, 2 allied health professionals). Thirty-five participants completed the post-course survey (response rate: 70%), with a balanced gender distribution (54% female). As detailed in Table 2, most participants were Italian nationals (89%), and two were paediatric emergency nurses.

**Table 2:** Demographics of the survey respondents

Variable	Survey Respondents (*n* = 35)	
	Absolute N°	%
*Gender*		
Male	15	43%
Female	19	54%
Non-binary/third gender	1	3%
*Nationality*		
Italian	31	89%
Non-Italian	4	11%
*Clinical Background*		
Medical students	9	26%
Residents	24	69%
Specialist	0	0%
Others	2	6%
*Participation in*		
Theoretical lectures	20	57%
Simulation and seminar	13	37%
Simulation only	2	6%

### Table 3 shows the aggragated results of the intercultural competencies accessed. Instructional quality

Residents rated instructional quality very highly (mean 4.78, 95% CI: 4.67–4.89). Exposure to diverse teaching styles, such as rapid-cycle simulations with immediate discussion of emotional and ethical challenges, enhanced understanding of cultural factors in care. In the U.S., intercultural norms are embedded in medical curricula from the undergraduate level, while in Italy, these skills are typically acquired informally during clinical practice. This difference helps explain the flatter hierarchy and emphasis on psychological safety seen in the U.S. approach.

**Table 3:** Grouped scores for the survey responses

Topic/Domain	Mean	95% CI (±)	Lower CI	Upper CI
Cultural Awareness	4.72	0.06	4.66	4.78
Cultural Sensitivity	4.08	0.15	3.93	4.23
Cultural Competency	4.65	0.11	4.54	4.75

*“The sessions flattened hierarchies; everyone contributed*,* which made decision-making in the simulation feel authentic and helped me speak up in the session.”*

### Cultural sensitivity

Motivation to apply ethical considerations and show respect towards diverse values was scored with 4.08 (95% CI [3.93–4.23]). Residents emphasized the value of learning strategies that explicitly addressed cultural diversity in paediatric care, including attention to racial, socioeconomic, and linguistic disparities rarely discussed in local curricula:

*“I will now consider potential differences in treatment among ethnic groups and aim to provide equitable care.”*

### Cultural competency

Residents reported strong learning gains in cultural competency (M = 4.65, 95% CI [4.54–4.75]). Qualitative feedback highlighted that exposure to diverse cultural perspectives and structured debriefings helped translate cultural concepts into practical skills for patient care.

### Cultural awareness

Participants reported a strong positive impact of the course on cultural awareness. “Appreciation of cultural diversity” received a mean score of 4.58 (95% CI: 4.32–4.84). Overall, residents reported enhanced awareness (M = 4.72, 95% CI [4.66–4.78]) of how cultural backgrounds influence patient care. Qualitative data confirmed that structured debriefings and culturally themed simulations (e.g., refugee patients, language barriers) helped translate abstract concepts into actionable strategies:

*“Faculty brought up disparities such as racial, socioeconomic, etc., but this is rarely mentioned locally.”*

### Professional development and global collaboration

Participants reported increased interest in pursuing international training and in maintaining professional networks abroad. Over half expressed motivation to continue engagement in global learning initiatives:

*“Connecting with faculty from other countries gave me new ideas for improving care in multicultural settings and inspired me to consider exchanges or electives abroad.”*

These findings suggest that internationalized faculty exchange programs can stimulate long-term professional aspirations and foster a global outlook, beyond immediate skill acquisition.

### Barriers and challenges

Barriers to learning were minimal, with language challenges reported as negligible. Minor difficulties with unfamiliar teaching methods were mitigated through bilingual facilitation and structured debriefings.

### Faculty perspectives

All visiting faculty agreed that the program enhanced residents’ cultural awareness and engagement, emphasizing the reciprocal nature of the exchange:

*“While we shared our simulation methods, we also learned from Humanitas’ approach to integrating deep knowledge into residency training and observed how residents critically appraise scenarios based on this strong foundation. The exchange was truly reciprocal.”*

Faculty concerns focused on the sustainability and scalability of international collaborations, rather than on content or implementation.

## Discussion

The results of this study demonstrate that internationalized, simulation-based training can meaningfully enhance intercultural learning, professional development, and global collaboration among residents in paediatric emergency medicine. Participants reported strong gains in cultural awareness, sensitivity, and competency, with mean scores consistently above 4.5 across domains. Qualitative feedback reinforced these findings, highlighting the practical translation of abstract cultural concepts into actionable strategies within clinical and team-based contexts. This supports the hypothesis that structured exposure to diverse teaching styles and intercultural frameworks, facilitated through faculty mobility or high-fidelity simulations, can provide tangible educational benefits beyond traditional curricula [20, 21].

Our findings align with previous research emphasizing the importance of cultural competence in medical education as a core professional skill rather than an optional supplement [8, 22]. Notably, the structured nature of the Paediatric EmergenSIMs Pathway, combining simulation, deliberate debriefing, and international faculty exchanges, appears to bridge a critical gap in Italian residency training, where intercultural skills are typically learned informally. The flattening of hierarchical structures and promotion of psychological safety, consistently highlighted by participants, reflects pedagogical practices common in U.S. medical education, underscoring the value of cross-national collaboration in shaping learners’ professional behaviours [17].

Beyond its intercultural benefits, this study underscores the unique power of simulation as a pedagogical tool in health professions education. Simulation provides a psychologically safe environment where learners can actively engage with complex, real-world scenarios, including those involving cultural, ethical, and communication challenges, without risk to patients [23, 24]. In our program, simulation was not only a vehicle for technical training but also a tool for developing cultural humility, teamwork, and adaptive expertise; qualities that are increasingly vital in today’s diverse and globalized healthcare settings. By integrating intercultural elements into high-fidelity simulation, we offer a replicable model for advancing both clinical and cultural competence, supporting the broader mission of preparing health professionals for effective practice in multicultural environments.

Finally, the program demonstrated broader professional and global benefits. Participants expressed increased motivation to pursue international electives and maintain professional networks abroad, suggesting that exposure to intercultural and simulation-based learning can stimulate long-term engagement with global health initiatives. This finding confirms prior literature suggesting that international faculty mobility contributes not only to immediate learning outcomes but also to the cultivation of a global mindset and professional adaptability [8, 25, 26]. Faculty perspectives reinforced the reciprocal nature of such exchanges, indicating that both hosts and visitors benefit through bidirectional knowledge transfer, a critical consideration for sustainable international collaborations [27].

Despite these positive outcomes, several challenges and considerations emerged. Sustainability and scalability remain central concerns, particularly given the resource-intensive nature of faculty mobility and high-fidelity simulations. These findings highlight the need for careful planning, institutional commitment, and integration of intercultural objectives into broader curricular frameworks to maximize the return on investment [14]. This study also addresses a notable gap in the literature: the lack of a structured framework for evaluating intercultural outcomes linked to international faculty mobility [28]. By combining quantitative surveys with qualitative reflections, our mixed-methods approach provides both measurable and contextualized insights into learning gains. Such an approach could inform the development of standardized metrics for assessing intercultural competence, enabling broader adoption and rigorous evaluation of internationalized medical education programs.

### Limitations

Several limitations should be noted. The single-centre nature of this study and the US-only faculty cohort limit generalizability; however, the intent was to explore proof-of-concept benefits, laying the groundwork for broader, multicentre European projects. Additionally, outcomes were based on perceived learning rather than objective behavioural change, highlighting the need for standardized frameworks to measure intercultural competencies and clinical application. No long-term follow-up was conducted, and there was no comparative group, preventing attribution of observed gains solely to the Paediatric EmergenSIMs Pathway. Future studies should include multi-centre designs, longitudinal assessment, objective outcomes, and control groups to strengthen evidence on effectiveness.

## Conclusion

This study demonstrates that structured, internationalized, simulation-based education can effectively enhance residents’ cultural awareness, sensitivity, and competence, while fostering interest in global collaboration and professional development. The program highlights the value of combining faculty mobility with high-fidelity simulations to translate intercultural concepts into actionable skills within clinical and team-based contexts. By identifying measurable aspects of IoME and using both qualitative and quantitative methods, this study helps define and demonstrate the value of internationalisation, laying groundwork for future program development and assessment in postgraduate training.

## Supplementary Information

Supplementary Material 1.

Supplementary Material 2.

## Data Availability

The datasets used and/or analysed during the current study are available from the corresponding author upon reasonable request.

## References

[1] Wu A, Choi E, Diderich M, Shamim A, Rahhal Z, Mitchell M, et al. Internationalization of medical education — motivations and formats of current practices. Med Sci Educ. 2022. 10.1007/s40670-022-01553-6. 35493984 10.1007/s40670-022-01553-6 PMC9044376

[2] Wijnen-Meijer M. Implications of internationalisation of medical education. BMC Med Educ. 2023. 10.1186/s12909-023-04630-5. 37679717 10.1186/s12909-023-04630-5 PMC10486115

[3] Wu A. Reshaping internationalization of medical education in 2023. BMC Med Educ. 2023. 10.1186/s12909-023-04374-2. 37221527 10.1186/s12909-023-04374-2 PMC10204034

[4] Mohiyeddini C. The imperative for cross-cultural medical education in globalized healthcare. Front Psychol. 2024. 10.3389/fpsyg.2024.1326723. 39118850 10.3389/fpsyg.2024.1326723 PMC11306068

[5] Lucza L, Martos T, Sallay V, Simon T, Weiland A, Vermeir P, et al. Profiles of intercultural sensitivity of healthcare students: a person-centred approach. Int J Med Educ. 2024;15:113–23. 10.5116/ijme.66dd.beb3. 39348553 10.5116/ijme.66dd.beb3 PMC11687379

[6] Stütz A, Green W, McAllister L, Eley D. Preparing medical graduates for an interconnected world: current practices and future possibilities for internationalizing the medical curriculum in different contexts. J Stud Int Educ. 2015. 10.1177/1028315314536991.

[7] Betancourt JR, Green AR, Carrillo JE, Ananeh-Firempong O. Defining cultural competence: a practical framework for addressing racial/ethnic disparities in health and health care. Public Health Rep. 2003;118:293–302. 10.1016/S0033-3549(04)50253-4. 12815076 10.1016/S0033-3549(04)50253-4 PMC1497553

[8] Shen W, Xu X, Wang X. Reconceptualising international academic mobility in the global knowledge system: towards a new research agenda. High Educ (Dordr). 2022;84:1317–42. 10.1007/s10734-022-00931-8. 36211225 10.1007/s10734-022-00931-8 PMC9527386

[9] Waterval DGJ, Frambach JM, Driessen EW, Scherpbier AJJA. A literature review of crossborder curriculum partnerships. J Stud Int Educ. 2015. 10.1177/1028315314533608.

[10] Purdy E, Alexander C, Caughley M, Bassett S, Brazil V. Identifying and transmitting the culture of emergency medicine through simulation. AEM Educ Train. 2019;3:118–28. 10.1002/aet2.10325. 31008423 10.1002/aet2.10325 PMC6457353

[11] Walkowska A, Przymuszała P, Marciniak-Stępak P, Nowosadko M, Baum E. Enhancing cross-cultural competence of medical and healthcare students with the use of simulated patients—a systematic review. Int J Environ Res Public Health. 2023;20:2505. 10.3390/ijerph20032505. 36767872 10.3390/ijerph20032505 PMC9916152

[12] Ebm C, Sarti R, Panico P, Pagliotta M, Vinci V, Oldani S. Enhancing compassion in medical education - a comparative study of the efficacy of clinical clerkships versus simulation-based training methodologies. BMC Med Educ. 2025;25:181. 10.1186/s12909-025-06687-w. 39905468 10.1186/s12909-025-06687-w PMC11796239

[13] Ebm C, Istrate M, Van Gelder F, Szőllősi GJ, Alexandre J, Azoulay E, et al. Return on investment of rapid ICU workforce upskilling: an economic and cost-effectiveness analysis. Intensive Care Med. 2025;51:1453–61. 10.1007/s00134-025-08033-6. 40689971 10.1007/s00134-025-08033-6 PMC12317895

[14] Barker LT, Meguerdichian M, Walker K, Janssens S, Szabo RA, Lopez C, et al. Value-based simulation in healthcare: a new model for metrics reporting. Adv Simul. 2025;10:41. 10.1186/s41077-025-00368-w. 10.1186/s41077-025-00368-w PMC12305902 40721827

[15] Rukadikar C, Mali S, Bajpai R, Rukadikar A, Singh AK. A review on cultural competency in medical education. J Family Med Prim Care. 2022;11:4319–29. 10.4103/jfmpc.jfmpc_2503_21. 36352918 10.4103/jfmpc.jfmpc_2503_21 PMC9638640

[16] Brouwer E, Frambach J. Solutionism across borders: sorting out problems, solutions and stakeholders in medical education internationalisation. Med Educ. 2021. 10.1111/medu.14384. 33001479 10.1111/medu.14384 PMC7756551

[17] Wu A, Leask B, Choi E, Unangst L, de Wit H. Internationalization of medical education—a scoping review of the current status in the United States. Med Sci Educ. 2020. 10.1007/s40670-020-01034-8. 32837797 10.1007/s40670-020-01034-8 PMC7406216

[18] Robertson AC, Fowler LC, Niconchuk J, Kreger M, Rickerson E, Sadovnikoff N, et al. Application of kern’s 6-Step approach in the development of a novel anesthesiology curriculum for perioperative code status and goals of care discussions. J Educ Perioper Med. 2019;21:E634. 31406705 PMC6685461

[19] Ebm C, del Pozo C, Barbarello A, Poli G, Brusa S. Unleashing excellence: using a project management approach to effectively implement a simulation curriculum to improve residents’ preparedness. BMC Med Educ. 2024. 10.1186/s12909-024-05166-y. 38438940 10.1186/s12909-024-05166-y PMC10913544

[20] Wojcieszek A, Kurowska A, Wróbel A, Bodys-Cupak I, Kamińska A, Majda A. Analysis of high-fidelity simulation effects and their connection with educational practices in early nursing education. BMC Nurs. 2025;24:457. 10.1186/s12912-025-03077-x. 40275323 10.1186/s12912-025-03077-x PMC12023673

[21] Galan-Lominchar M, Roque IM-S, Cazallas C, del Mcalpin C, Fernández-Ayuso R, Ribeiro D. Nursing students’ internationalization: virtual exchange and clinical simulation impact cultural intelligence. Nurs Outlook. 2024;72:102137. 10.1016/j.outlook.2024.102137. 38340388 10.1016/j.outlook.2024.102137

[22] Wu A, Leask B, Noel G, De Wit H. It is time for the internationalization of medical education to be at home and accessible for all. Acad Med. 2021. 10.1097/ACM.0000000000004201. 34108376 10.1097/ACM.0000000000004201 PMC8378433

[23] Mitchell AM, Fioravanti M, Founds S, Hoffmann RL, Libman R. Using Simulation to Bridge Communication and Cultural Barriers in Health Care Encounters: Report of an International Workshop. Clin Simul Nurs. 2010;6:e193–8. 10.1016/j.ecns.2009.10.001.

[24] Isaksson J, Krabbe J, Ramklint M. Medical students’ experiences of working with simulated patients in challenging communication training. Adv Simul. 2022;7:32. 10.1186/s41077-022-00230-3. 10.1186/s41077-022-00230-3 PMC9552443 36217210

[25] Stohl M. We have met the enemy and he is us: the role of the faculty in the internationalization of higher education in the coming decade. J Stud Int Educ. 2007. 10.1177/1028315307303923.

[26] Oliveira Hashiguchi L, Conlin M, Roberts D, McGee K, Marten R, Nachuk S, et al. Enabling cross-country learning and exchange to support universal health coverage implementation. Health Policy Plan. 2024;39:i125–30. 10.1093/heapol/czad097. 38253439 10.1093/heapol/czad097 PMC10803195

[27] Brouwer E, Driessen E, Mamat NH, Nadarajah VD, Somodi K, Frambach J. Educating universal professionals or global physicians? A multi-centre study of international medical programmes design. Med Teach. 2020. 10.1080/0142159X.2019.1676885. 31630598 10.1080/0142159X.2019.1676885

[28] Arruzza E, Chau M. The effectiveness of cultural competence education in enhancing knowledge acquisition, performance, attitudes, and student satisfaction among undergraduate health science students: a scoping review. J Educ Eval Health Prof. 2021;18:3. 10.3352/jeehp.2021.18.3. 33621460 10.3352/jeehp.2021.18.3 PMC8089465

