# Supplementary material for: Preparing Italian residents for global medical practice: the role of internationalization in education
Source: Adv Simul (Lond). 2025 Nov 25;10:61. doi: 10.1186/s41077-025-00394-8 (PMC12645667; doi:10.1186/s41077-025-00394-8)
Supplement: Supplementary file 1 — Supplementary Material 1. [file 41077_2025_394_MOESM1_ESM.docx]

**Examples of Simulation cases**

**Simulation Case: Febrile Seizure in a Child of a Migrant Worker**

**Scenario:**
A 3-year-old child of Romanian migrant workers is brought in after a first febrile seizure. The parents are concerned about hospitalization due to work and financial constraints.

**Intercultural Elements:**

- Socioeconomic and access-to-care challenges
- Parental expectations and fears about the healthcare system
- Navigating consent and shared decision-making across cultures

**Learning Objectives:**

- Address socioeconomic and cultural factors influencing care decisions
- Practice shared decision-making with families from diverse backgrounds
- Foster psychological safety and trust in cross-cultural encounters

**Simulation Case: Cross-Cultural Pediatric Handover**

**Scenario:**
A pediatric patient is being transferred from the emergency department (led by a U.S. visiting physician) to the inpatient ward (led by an Italian resident team). The handover occurs in English, but some team members are non-native speakers. The patient’s family is present and speaks only Romanian.

**Intercultural Elements:**

- Language barriers and use of interpreters
- Differences in communication styles (direct vs. indirect, hierarchical vs. flat)
- Cultural expectations about family involvement and decision-making
- Clarifying roles and responsibilities across teams

**Learning Objectives:**

- Practice clear, structured handover using SBAR (Situation, Background, Assessment, Recommendation)
- Identify and address misunderstandings due to language or cultural differences
- Demonstrate culturally sensitive communication with both colleagues and the patient’s family
- Foster psychological safety, encouraging questions and clarifications from all team members
