# Supplementary material for: Preparing Italian residents for global medical practice: the role of internationalization in education
Source: Adv Simul (Lond). 2025 Nov 25;10:61. doi: 10.1186/s41077-025-00394-8 (PMC12645667; doi:10.1186/s41077-025-00394-8)
Supplement: Supplementary file 2 — Supplementary Material 2. [file 41077_2025_394_MOESM2_ESM.docx]

**Interview Guide for the qualitative interviews**

**1. Background and Motivation**

- Can you briefly describe your role and training background?
- What motivated you to join this course?

**2. Learning Experience**

- How would you describe your experience with the intercultural aspects of the course?
- Were there specific moments that made you reflect on cultural differences in clinical practice?

**3. Impact and Application**

- Has the course influenced your approach to culturally sensitive care?
- Do you feel more confident in handling intercultural situations in your clinical work?

**4. Faculty and Environment**

- How did the faculty’s international background contribute to your learning?
- Did the diversity of perspectives enhance your understanding?

**5. Suggestions and Barriers**

- Positive impressions?
- What improvements would you suggest for future editions?
- What barriers did you encounter?
